# A142 APPROPRIATENESS OF POST-ENDOSCOPY CARE IN PATIENTS PRESENTING WITH FOOD BOLUS IMPACTIONS OVERNIGHT: A POPULATION-BASED MULTICENTER COHORT STUDY

**DOI:** 10.1093/jcag/gwac036.142

**Published:** 2023-03-07

**Authors:** H Guo, P Hamilton, E Enns, M Gupta, C Andrews, Y Nasser, A Bredenoord, E Dellon, C Ma

**Affiliations:** 1 Division of Gastroenterology and Hepatology; 2 Department of Medicine, University of Calgary; 3 Department of Medicine, Alberta Health Services; 4 Snyder Institute for Chronic Diseases, University of Calgary, Calgary, Canada; 5 Department of Gastroenterology and Hepatology, Academic Medical Center, Amsterdam, Netherlands; 6 Division of Gastroenterology and Hepatology, University of North Carolina School of Medicine, Chapel Hill, United States; 7 Department of Community Health Sciences, University of Calgary, Calgary, Canada

## Abstract

**Background:**

Appropriate management of esophageal food bolus impactions includes endoscopic evaluation and follow-up for potential underlying esophageal pathology. Patients who present with impactions at night may not receive optimal long-term post-endoscopy care due to patient-, physician-, or system-related factors.

**Purpose:**

We aimed to evaluate the appropriateness of care for patients who present with food bolus impactions after regular daytime hours.

**Method:**

We conducted a retrospective, population-based, multi-center cohort study of adult patients undergoing endoscopy for food impaction between 19:00-06:59 from 2016-2018 in the Calgary Health Zone, Canada. Appropriate post-endoscopy care was defined by a composite of a follow-up clinic visit, repeat endoscopy, other appropriate investigations (e.g., manometry), or appropriate medical treatment (e.g., proton pump inhibitor). Predictors of inappropriate care were assessed using multivariable logistic regression, expressed as adjusted odds ratios (aOR) with 95% confidence intervals (CI).

**Result(s):**

A total of 323 patients underwent an after-hours or overnight endoscopy for food bolus impaction. 25.4% (82/323) of patients did not receive appropriate post-endoscopy care. Predictors of inappropriate care included rural residence (aOR 2.66 [95% CI: 1.18-6.01], p=0.02), first food bolus presentation (aOR 2.38 [95% CI: 1.04-5.44], p=0.04), and absence of a specific pathology during the index procedure (aOR 3.01 [95% CI: 0.97-9.29], p=0.05), suggesting a potential association with clinician cognitive bias. Among patients who were followed, 18.9% (35/185) had a change in the original diagnosis.

**Image:**

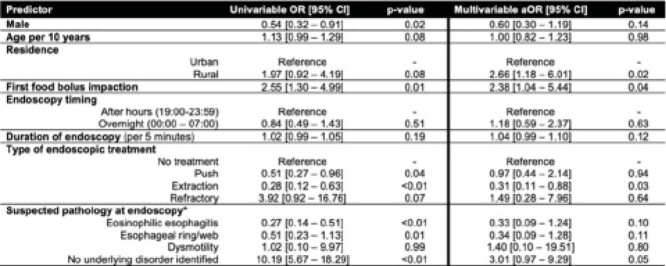

**Conclusion(s):**

One quarter of patients presenting with a food bolus impaction at night do not receive appropriate post-endoscopy care. System-based interventions should target this high-risk population as the diagnosis and management may change with follow-up.

**Please acknowledge all funding agencies by checking the applicable boxes below:**

None

**Disclosure of Interest:**

None Declared

